# Examining Variable Domain Orientations in Antigen Receptors Gives Insight into TCR-Like Antibody Design

**DOI:** 10.1371/journal.pcbi.1003852

**Published:** 2014-09-18

**Authors:** James Dunbar, Bernhard Knapp, Angelika Fuchs, Jiye Shi, Charlotte M. Deane

**Affiliations:** 1 Department of Statistics, University of Oxford, Oxford, United Kingdom; 2 F. Hoffmann-La Roche Ltd, Pharma Research and Early Development, Informatics, Penzberg, Germany; 3 Informatics, UCB Pharma, Slough, United Kingdom; Bar Ilan University, Israel

## Abstract

The variable domains of antibodies and T-Cell receptors (TCRs) share similar structures. Both molecules act as sensors for the immune system but recognise their respective antigens in different ways. Antibodies bind to a diverse set of antigenic shapes whilst TCRs only recognise linear peptides presented by a major histocompatibility complex (MHC). The antigen specificity and affinity of both receptors is determined primarily by the sequence and structure of their complementarity determining regions (CDRs). In antibodies the binding site is also known to be affected by the relative orientation of the variable domains, VH and VL. Here, the corresponding property for TCRs, the Vβ-Vα orientation, is investigated and compared with that of antibodies. We find that TCR and antibody orientations are distinct. General antibody orientations are found to be incompatible with binding to the MHC in a canonical TCR-like mode. Finally, factors that cause the orientation of TCRs and antibodies to be different are investigated. Packing of the long Vα CDR3 in the domain-domain interface is found to be influential. In antibodies, a similar packing affect can be achieved using a bulky residue at IMGT position 50 on the VH domain. Along with IMGT VH 50, other positions are identified that may help to promote a TCR-like orientation in antibodies. These positions should provide useful considerations in the engineering of therapeutic TCR-like antibodies.

## Author Summary

The immune system needs to be able to sense molecules that might be harmful to the organism. Such harmful molecules are known as antigens. Two classes of receptor proteins that mediate antigen recognition are antibodies and T-Cell receptors (TCRs). Antibodies are able to bind a diverse range of antigen shapes whilst TCRs are specialised to recognise a cell-surface protein, the pMHC. Antibodies that bind the pMHC are rarely created naturally. However, such TCR-like antibodies are of therapeutic importance. The binding regions of the TCR and the antibody have very similar three dimensional structures. Both consist of two independent units, domains, which associate and form the antigen binding site between them. This work examines how the two domains orientate with respect to one another in TCRs and antibodies. Our results show that the conformations that exist in TCRs and antibodies are distinct. Consequently it is difficult for an antibody to bind to a pMHC in the same way a TCR would. However, a similar conformation can be achieved in antibodies as in TCRs by the presence of certain amino-acids in the domain interface. This knowledge should aid the development of therapeutic TCR-like antibodies.

## Introduction

The immunoglobulin fold provides the scaffold for many different proteins with diverse a set of functions [1]. The immunoglobulin domain consists of two β sheets arranged in a sandwich motif. Many protein structures consist of multiple immunoglobulin-like domains. These domain types are particularly prevalent in the immune system of vertebrates.

A key task of the immune system is to specifically recognise molecules that are potentially pathogenic or foreign to the organism. Examples of components that enable this are B-cell receptors or, in their soluble form, antibodies. These are able to bind to antigens without the aid of other cellular machinery. The portion of the molecule that mediates antigen binding, the variable fragment (Fv), consists of two immunoglobulin domains, VH and VL.

In contrast, another component of the immune system, the T-cell receptor (TCR), binds only to peptide antigens and only when they are presented on the surface of a cell by the major histocompatibility complex (MHC). However, like the antibody, the TCR binds using its variable region that consists of two domains, Vα and Vβ, which are analogous to the antibody VL and VH domains.

Each of the variable domain types have three CDRs that are generally characterised as loop structures [2]–[4]. It is these six CDRs that are the primary determinants of antigen specificity and affinity. A fourth hyper-variable region is also found on the Vβ domain [5]. However, this is not thought to interact directly with the epitope. Each variable domain type is expressed from a different locus on the genome and is generated from a combination of genes. This allows large repertoires of potential receptor sequences to be generated. The antibody Vκ and Vλ (collectively VL) and the TCR Vα domain are generated from two gene types named variable (v) and joining (j). The antibody VH and TCR Vβ domains are built from v and j genes along with an additional gene type, the diverse (d) gene. The additional variability that the d gene brings is reflected in the sequences and structures of the third complementarity determining region (CDR) loop in VH and Vβ domains [6].

Recently, the way in which the antibody variable domains are orientated has been recognised as a determinant of antigen binding [7]. The variable domain orientation affects how the CDR loops are positioned relative to one another and therefore influences the geometry of the antigen binding site [8], [9]. Antibody humanization studies have found that mutations to framework residues distant from the binding site can act to regain the affinity of the original murine antibody [10]. This effect has been attributed to structural changes in the Fv of the antibody. One effect may be to change the relative orientation between the domains [8],[11],[12]. In addition to its biological importance, predicting the VH-VL orientation is also considered one of the challenges in modelling antibody structures [7], [13].

We and others have studied the geometry of the variable domain orientation in antibodies [14]–[17]. Distinct sets of orientations can be identified in antibodies and attributed to sequence differences on the domain-domain interface. One example of a functional difference between antibodies with distinct domain orientations is related to the size of the antigen they bind to [15]. Antigen size is also related to the level of variable domain orientation conservation found in sequence-identical structures [16]. Whilst antibodies can bind to a highly diverse set of antigenic shapes and sequences, TCR epitopes are relatively similar to one another. Therefore, although similar in function, the binding sites of the two molecule types are under different pressures. Antibodies must be versatile in binding to many different targets whilst the TCR antigen binding site can optimise to associate specifically with the shape of MHCs.

Although the diversity in potential T-Cell epitopes is much smaller than for antibodies, variation is present in the MHC class, allele, and the bound peptide [18]. MHC class I molecules (MHCI) are present on the surface of most cells. The binding groove consists of a single chain and is closed at both ends. This enables MHCI to present peptides of typically nine residues in length. MHCI peptides are of intracellular origin. The MHCI is attached by one trans-membrane region to the cell surface. In contrast, MHC class II molecules (MHCII) form their binding groove with two chains. The binding groove of MHCII is open at both ends allowing for peptides of lengths of 20 or more residues to be presented [19]. MHCII peptides are derived from extracellular proteins. The MHCII is attached with two transmembrane regions to the cell surface. Even within the same MHC class, sequence differences allow for a specific binding repertoire of peptides and TCRs [20]. Currently 8,124 human MHCI and 2,409 human MHCII alleles are known [21]. Despite these differences MHCs share the same overall fold with the peptide presented above an anti-parallel beta sheet floor and flanked by two kinked alpha helices. This allows the TCR binding site to be highly structurally conserved.

The relative similarity between T-Cell epitopes is also reflected in the structure of the complex formed between the TCR and MHC/peptide. Typically, the TCR is found to bind diagonally with respect to the MHC's peptide groove. This geometry of interaction is referred to as the canonical binding mode. Those cases where the TCR is positioned in a different orientation are known as non-canonical [22]. Although no strict definition for canonical association has been made, a number of TCRs that bind unusually have been found to be related to autoimmune responses [23], [24]. Ensuring a canonical binding mode may therefore be important for preventing the recognition of self- antigens.

Naturally, T-Cell and B-Cell receptors perform different functions in the immune system. An example of a non-natural interface between the two receptor types are TCR-like antibodies. These antibodies are specific for T-cell epitopes. Given that they bind to a component of the organism, they are not normally found naturally in the body. However, in diseases where T-cell functionality is inhibited, engineered TCR-like antibodies provide a method for delivering cytotoxic drugs and induction of infected cell apoptosis [25], [26]. An advantage of using a TCR-like antibody as a therapeutic over a soluble recombinant TCR [27] is their higher natural affinity to an antigen [28]. Subsequently, a lower level of affinity enhancement is required using techniques such as phage display. In addition, the serum half-life of an antibody is measured in days to weeks, whilst that of a TCR is of the order of hours [29].

Antibodies able to recognise T-cell epitopes must mimic a TCR and replicate their binding site properties to enable pMHC specificity. Producing TCR-like antibodies is a difficult procedure inhibited by the process of isolating the desired MHC/peptide epitope [26]. However, advances in phage-display technology have allowed the generation of such molecules for diagnostic and potential therapeutic purposes [30]–[32]. Successfully generated TCR-like antibodies share some similar pMHC-binding properties with TCRs that have the same specificity [32], [33]. For example, mutations to certain positions on the MHC disrupt both TCR and antibody binding [32]. However, previous comparisons between individual antibodies and TCRs have also reported that the receptors use different features to achieve pMHC specificity [32], [34], [35]. This suggests that antibodies do not necessarily mimic TCR canonical binding. The therapeutic importance of TCR-like antibodies motivates the investigation of how they may be engineered to exhibit improved MHC specificity. Whilst the TCR's CDR residues primarily mediate MHC recognition, the orientation between the TCR Vα and Vβ domains has previously been proposed to contribute in determining T-Cell epitope specificity [36].

In this study we investigate the Vβ-Vα domain orientations in TCR variable regions and compare them to the analogous structural space in antibodies. Functional reasons for differences in the two receptor types are proposed by analysing the influence of domain orientation on the structural properties of a TCR-MHC complex. Given that the orientation space is different we investigate which sequence positions may be contributing factors and suggest residues on the domain interface of antibodies that may induce a more TCR-like orientation.

## Materials and Methods

### Dataset

All X-Ray crystal structures containing a paired TCR α and β chain were extracted from the protein data bank (PDB) [37] using the international ImMunoGeneTics information system (IMGT) [38]. IMGT numbering was retained for each of the TCR variable domains. Those 92 structures with a resolution of less than 3 Å formed the full redundant dataset. This set contained 49 TCRs bound to MHCI, 15 bound to MHCII and 28 unbound. A sequence identity filter of 90% was applied over the TCR variable domains using cd-hit [39] to form a non-redundant set of 39 structures. Nineteen were bound to MHCI, 11 to an MHCII and 9 were unbound.

A non-redundant antibody dataset was created using the structural antibody database (SAbDab) [40]. Again, a sequence identity filter of 90% was applied to the full sequence of the variable domains using cd-hit. The non-redundant antibody set consisted of 441 structures. The IMGT DomainGapAlign tool [41] was used to apply IMGT numbering to the variable domains in each of the structures.

The structures in SAbDab were filtered to identify TCR-like antibodies bound to MHCs. A BLAST search [42] for the sequence of the MHCI and MHCII was performed using a database created from the antigens in SAbDab. Manual inspection of these top hits and a keyword search for phrases similar to “TCR like antibody” identified a total of 4 structures. One of the structures (3hae) is higher affinity mutant of another (3gjf) from the same experiment [33]. Therefore 3 cases were identified for TCR-like antibodies in complex with an MHC.

### Rationale for domain equivalence

To compare the variable domain orientations of antibodies and TCRs it is necessary to define which domains are equivalent in the two receptor types. Is VH equivalent to Vβ or Vα?

Comparing the mean sequence identity between the domain types showed that Vκ and Vλ (collectively VL) domains were more similar to both Vα and Vβ than VH was to either. Therefore, sequence identity alone provides no clear indication.

To compare the use of the VH-Vα/VL-Vβ or the VH-Vβ/VL-Vα equivalence we examined the residue conservation at individual IMGT positions in the sets of domains. To check the use of VL/Vα and VH/Vβ equivalence we identified IMGT positions where the VH and Vα domains have the same conserved residue whilst in VL domains a different conserved amino-acid is present. Positions such as these suggest that the alternative equivalence should be used. These positions were identified for both equivalences i.e. VH-Vβ/VL-Vα and VH-Vα/VL-Vβ. A position was deemed to be conserved if it had the same amino-acid in 50% or more sequences. Five positions support making VH-Vα/VL-Vβ equivalent whilst 10 positions support making VH-Vβ/VL-Vα equivalent. This equivalence is also supported by the similarity in the combination of genes that make up corresponding domains. Both Vβ and VH are generated from v, d and j genes whilst Vα and VL are built from just v and j genes. We therefore use the VH-Vβ/VL-Vα equivalence herein.

### Variable domain orientation root mean square deviation

The variable domain orientation root mean square deviation (RMSD) is a measure of the difference in orientation between two structures. As an example, the orientation RMSD between two TCR structures (Tx and Ty) is calculated as follows. Tx and Ty are structurally aligned using the Cα coordinates of their shared Vα domain framework positions. Tx's Vβ domain is then independently aligned to Ty's Vβ domain. The RMSD is calculated between Tx's Vβ domain in its native orientation and the transformed position. The same procedure is also performed using the Vβ domains for the initial alignment and calculating RMSD between Vα domains. The mean of these two values describes the difference in the relative orientation. All structural alignments were performed using the Biopython SVD superimposer [43].

The orientation RMSD was calculated for each pair of structures in the non-redundant antibody set, the non-redundant TCR set and between the sets. These measurements were used as distances between the structures allowing them to be clustered using a complete-linkage hierarchal clustering method [44].

### Applying the ABangle methodology to TCRs

Whilst relative measures of orientation differences such as the orientation RMSD give a magnitude to changes in pose they do not reveal *how* structures vary. The ABangle methodology was developed to characterise the orientation between the VH and VL domains in antibodies. It gives six absolute measures, five angles and a distance, to fully describe the orientation between the two domains. The same method can be applied to any set of structurally defined homologous domain pairs. The process is presented in detail in Dunbar *et al* 2013 [16] and can be summarised as:

1. Define the most structurally conserved positions for both domains (*coresets*).
2. Fit frames of reference through interface positions using coordinates from multiple structural alignment.
3. Define consensus (mean) structures for both domains.
4. Compute or choose the pivot axis about which to measure orientation.
5. Define six measures about the resulting coordinate system to describe orientation.

This process was followed for the TCR structures in our dataset. The *coreset* positions are listed in the supplementary material. To allow for direct comparisons to be made with antibodies, the pivot axis, C, was chosen to be the same as in the antibody ABangle work. However, the pivot axis was also calculated for TCRs and has been used in a separate study [45] to investigate variation in structural properties of the TCR-MHC/peptide complex using molecular dynamics simulations. Whilst the two axes are similar, the TCR-derived one lies closer to the centre of the domain-domain interface than the antibody derived one.

The resulting coordinate system allows for 6 absolute measures of orientation to be defined, five angles and a distance. The distance, dc, is the length of C. HL describes a torsion angle of one domain with respect to the other and is similar to the packing angle defined by Abhinandan et al [14]. HC1 and LC1 are angles that describe the tilt of one domain towards the other. HC2 and LC2 describe twisting-like differences between the orientations of variable domain structures. In each of these cases the ABangle antibody nomenclature has been retained.

### The MHC-TCR docking angle

TCRs are often observed to bind to the MHC in a diagonal mode with respect to the peptide binding groove. Qualitative observations have labelled this association geometry as the canonical binding mode. The binding geometry can also be described quantitatively using the MHC-TCR docking angle [46]. This is defined as the angle between the major axis of the peptide and the vector between the Cα atoms of the interface cysteines (IMGT position 104) on the Vα and Vβ domains. We calculated the docking angle for both TCRs and antibodies bound to an MHC. A canonical angle for TCRs is defined as being between 40° and 85°, a range similar to that found by previous studies [22],[47].

### Measuring the effect of variable domain orientation in a TCR-MHC complex

The effect that changing the Vβ-Vα orientation has on the structure of a TCR-MHC complex was investigated. A single TCR-MHC-peptide complex was chosen (PDB 1mi5 [48]) as the native structure. The MHC is of class I and presents a peptide 9 residues in length from the Epstein Barr Virus. Both the MHC and TCR are human in origin and the structure is solved at a resolution of 2.5 Å. The docking angle of the TCR-MHC is 51.4° and is therefore considered to be in the canonical range. A set of 20 non-redundant structures were chosen from across the antibody orientation space (Table S1 in Text S1). Similarly, a set of 20 structures was sampled from the non-redundant TCR dataset (Table S2 in Text S1). These two sets are referred to as the antibody and TCR decoy sets respectively.

The native complex was compared to structures made when the Vβ-Vα orientation was changed to assume the poses in the TCR and antibody decoy sets. The following protocol was used to change the Vβ-Vα orientation.

**Figure 1:**
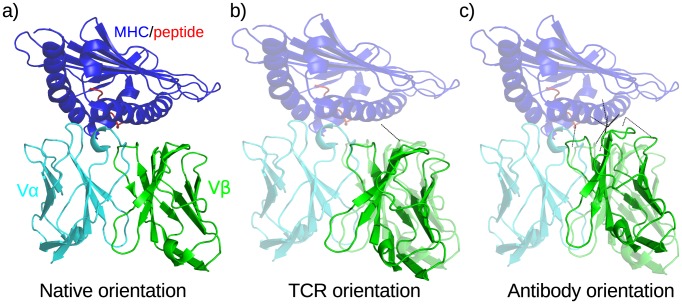
Changing the Vβ-Vα orientation of a TCR/MHC-peptide complex. a) The native complex 1mi5. No residue clashes are observed between the TCR and the MHC-peptide. b) The complex with the TCR placed in the Vβ-Vα orientation of another TCR structure. A single clash (black line) is identified between the Vβ domain and the MHC. c) The complex with the TCR placed in the VH-VL orientation of an antibody. Multiple residue clashes, shown by black lines, are found between the Vβ domain and the MHC.

Given a decoy structure, the VL domain (or Vα domain for TCR decoys) was aligned to the Vα domain of the native complex (Figure 1a). This structural alignment was performed using the shared framework positions of the two domains. The native Vβ domain was then transformed independently to assume the resulting pose of the decoy VH domain (or Vβ domain for TCR decoys). Thus, the Vα domain is in a native position relative to the MHC-peptide complex whilst the Vβ domain inherits the orientation of the decoy (Figure 1b,c).

After remodelling the residue side chains with Scwrl4 [49], clashes were identified between the Vβ domain in its decoy position and the MHC/peptide chains using the program Molprobity [50]. Any pair of residues with a Cβ –Cβ atomic distance of less than 7 Å were also recorded. In addition, the DOPE score [51] of the entire complex was calculated to assess the energetic change in modifying the domain orientation. The process was then repeated in an analogous way to change the Vα position to assume the decoy orientation whilst maintaining a native Vβ pose.

## Results

### Vβ-Vα orientations are different from VH-VL orientations

**Figure 2:**
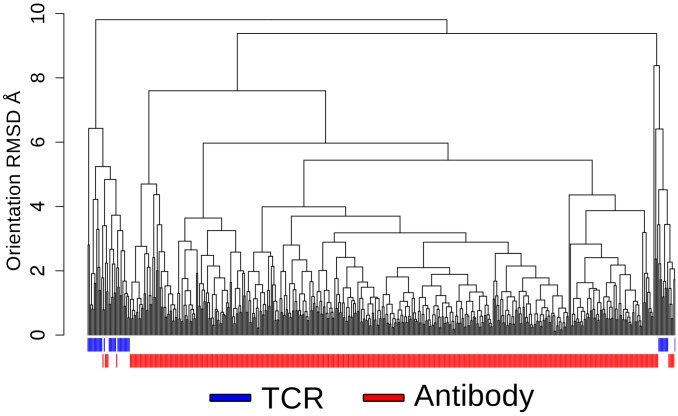
Clustering of the antibody and TCR structures by their relative orientation RMSD. The two types of structure fall largely into different clusters.

Antibody and TCR structures were clustered based on their orientation RMSD (Figure 2). The TCR and antibody structures fall into separate clusters with very little mixing of the two types. This demonstrates a difference in the variable domain orientations of TCRs and antibodies. The orientations of the TCRs alone fall into two distinct clusters. One might suspect that this may be related to MHC type or species. However, neither cluster has a strong bias for either property. Furthermore, no clear general characteristic can be identified to separate TCRs with these different conformations. In general, TCR binding orientation appears to show little relationship to Vβ-Vα orientations, however the scarcity of non-canonical binding examples makes it hard to draw any specific conclusions.

Whilst the RMSD measure shows that the orientations of antibodies and TCRs have distinct conformations it does not show us how the structures differ. We therefore used the ABangle methodology to identify structural differences.

### ABangle measures reveal how antigen receptors differ

**Figure 3:**
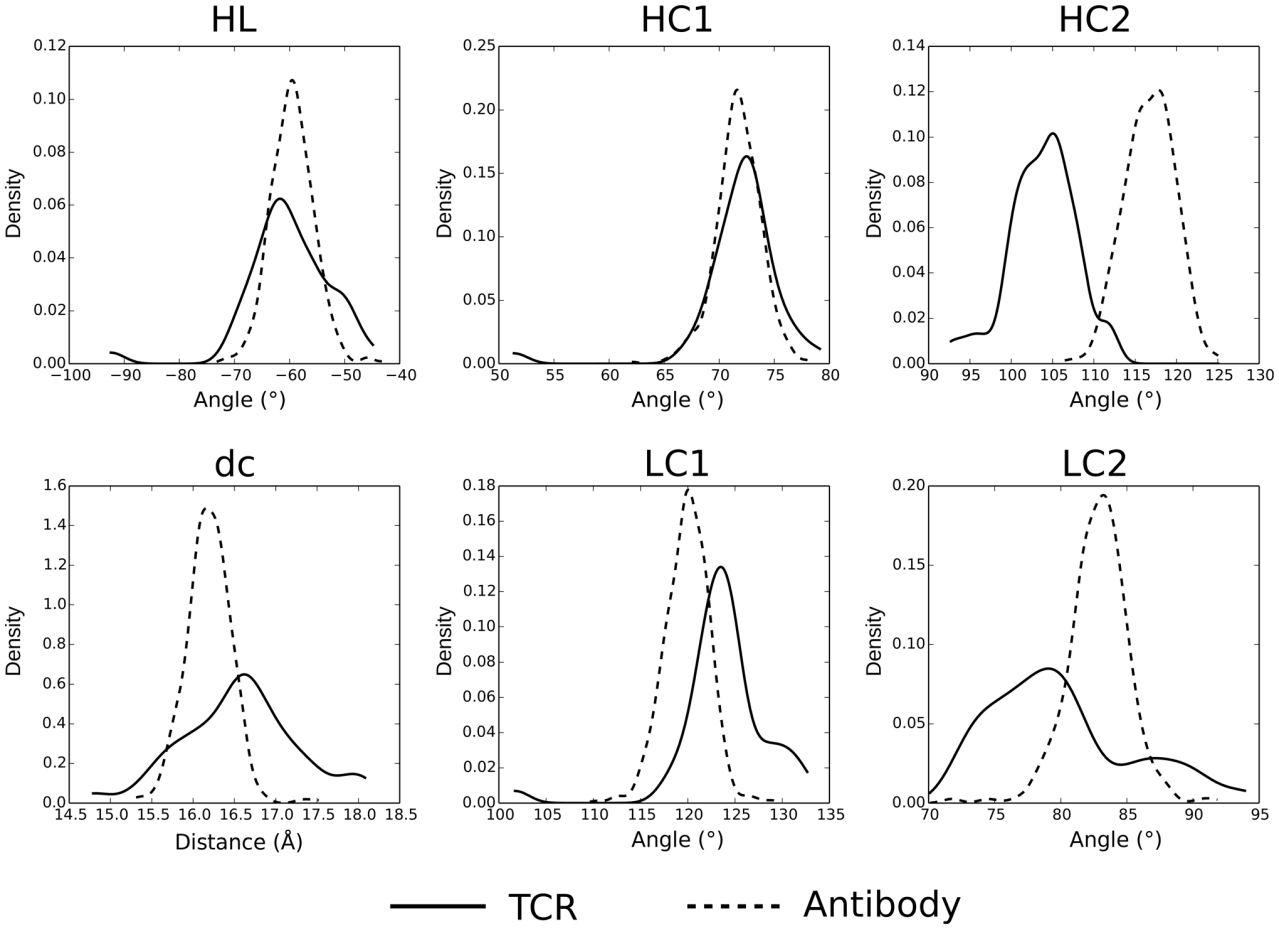
The ABangle orientation measures for antibodies and TCRs. The receptor types are found to have different orientations. The difference is best characterised by the HC2 angle. This corresponds to a twist of the VH or Vβ domain with respect to the VL or Vα domain respectively.

**Figure 4:**
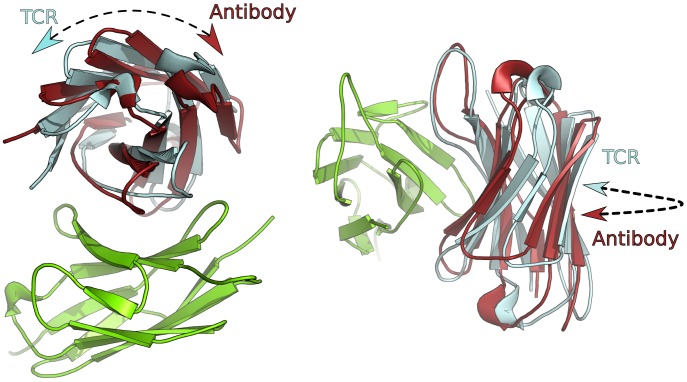
Two views showing the difference in variable domain orientation between antibodies and TCRs. The VH domain (red) and VL domain (green) of the antibody structure 3hzk is shown in its native orientation. The same antibody is also shown in the orientation assumed from the TCR structure 3qiu. In this case the VH domain is shown in cyan. The difference in variable domain orientation can be described as a twist of one domain with respect to the other and is best characterised using our HC2 twist angle.

ABangle's six orientation measures were calculated for each structure in the dataset. Figure 3 shows the distributions for both antibodies and TCRs. A similar magnitude of variation is observed in the orientations of the two sets of structures. However, as with the RMSD measure of orientation, a clear difference in orientation preference is seen between the two structure sets. This is best characterised in the HC2 angle where the distributions are significantly different (Kolmogorov–Smirnov test p-value 2.2×10^−16^). In fact, antibodies are almost never observed to reach the extreme HC2 orientation seen in TCRs. Two of the other orientation angles, LC1 and LC2 are also significantly different (p-values 7.1×10^−12^ and 8.6×10^−10^ respectively) whilst the two receptor types do not have significantly different HL torsion angles or HC1 bend angles. The distributions of the dc length are significantly different with a p-value of 3.46×10^−8^. The difference in orientation between antibodies and TCRs can be best described as a twisting-like change of the variable domains with respect to one another. Figure 4 demonstrates such a structural difference in orientation between the antigen receptor types.

### Antibody orientations are incompatible with binding in a TCR-like mode

For such similar molecules in terms of their function and domain structure, it is somewhat surprising that TCRs and antibodies have such different orientations. As discussed previously, TCRs bind specifically to peptide antigens only when they are presented by MHCs on the surface of a cell. In comparison, antibodies are not restricted in the same way and bind to a far more varied set of antigenic shapes.

To investigate whether the variable domain orientation is likely to affect function we calculated the influence that changing the Vβ-Vα orientation had on the structure of a single TCR-MHC complex PDB 1mi5). Two sets of orientation decoys were used, one from antibody structures and the other from other TCR structures (for full details see the materials and methods section).

For both of the decoy sets, the number of Vα-MHC/peptide residue clashes and number of Vβ-MHC/peptide residue clashes were counted using Molprobity. No clashes were observed between either domain and the MHC/peptide chains in the native complex. For the TCR decoy set medians of 3.5 and 5 clashes were found for the Vα and Vβ domains respectively (4 and 8.5 before side chain rearrangement). In comparison, medians of 5 and 8 clashes were induced for the equivalent domains using the antibody decoys (10 and 14 before side chain rearrangement). A Mann-Whitney U test found the increase in the total number of clashes to be statistically significant (p-value 0.005). The increased number of clashes between the proteins suggests that antibody orientations are incompatible with binding in a canonical TCR-like mode. For the native complex and the re-orientated complex sets, the mean Cβ-Cβ contact distances were calculated. The contact matrices for each set are shown in Supplementary Figure S5 in Text S1.

The difference between the antibody-orientated complexes and the TCR-orientated complexes can also be measured using the energetics of the structures. Supplementary Figure S1 in Text S1 shows the distributions of the DOPE score relative to the native state. Making the assumption that the native state approximates an energy minimum, we find that both sets of complexes are transformed to less favourable, higher energy states represented by higher DOPE scores. However, the set of complexes with orientations assumed from other TCRs are found to be significantly closer to the native state score than those with antibody orientations.

These results suggest that binding in a TCR-like mode requires the receptor to have a TCR-like variable domain orientation. Although the Vβ-Vα orientation is at least as variable as the VH-VL orientation, TCR conformations are generally compatible with binding to the MHC in a canonical mode. In contrast, the space of antibody orientations is such that the canonical mode becomes more difficult to obtain. Despite this observation, antibodies may be engineered such that they bind to MHC-peptide epitopes. These molecules are known as TCR-like antibodies.

### Orientations of TCR-like antibodies

The orientations of each of the TCR-like antibodies found in SAbDab were calculated using the ABangle methodology. Supplementary Figure S2 in Text S1 shows where each of these lie compared to the background antibody distribution. There appears to be no preference for their variable domain orientation. However, one structure, PDB 3cvh, has an orientation that is considered extreme for an antibody. It has an HC2 angle that leans towards the domain orientations of TCRs.

To determine whether these TCR-like antibodies behave similarly to TCRs we investigated the geometry of the complex they form with the MHC using the docking angle (see materials and methods). Most of the antibody complexes do not bind in a canonical manner (Table 1). Instead, they bind diagonally but appear to use residues in the VL domain in the same way as a TCR uses the Vβ residues. Likewise the VH domains sit in a similar position to Vα domains in the canonical TCR-MHC complexes.

**Table 1:** The docking angle for each TCR-like antibody/MHC complex.

PDB	Heavy Chain	Light Chain	Docking Angle
3cvh	H	L	48.9°
3cvh	Q	R	49.3°
3gjf	H	L	122.3°
3gjf	M	K	125.1°
3hae	H	L	124.7°
3hae	I	G	124.9°
3hae	O	N	126.2°
3hae	T	S	123.6°
1w72	H	L	139.6°
1w72	I	M	140.3°

Only one of the TCR-like antibodies, 3cvh, binds in a similar mode to the TCR structures and with the expected equivalence. As discussed above, 3cvh, is the only antibody that had an variable domain orientation that approached that seen in the TCR space. Although only a single example, this structure suggests that in order to be able bind in a TCR-like mode to the MHC, a TCR-like variable domain orientation should be promoted.

### Factors for promoting a TCR-like VH-VL orientation

Given that variable domain orientation may be related to the functions of antibodies and TCRs, we investigated which factors give rise to the structural differences between them. The type of antigen an antibody binds does not determine its absolute VH-VL orientation [16] (Figure S6 in Text S1). Consequently, peptide-binding antibodies are no more similar in orientation to TCRs than general antibodies. The largest sequence variation in both receptor types occurs in the CDRs. Insertions also occur in these regions and cause variation in CDR loop structures length. The CDR3 in each domain type is likely to be the most influential loop as it makes the most inter-chain contacts.

Supplementary Figures S3a and S3b in Text S1 show the length distributions of CDR3 in VL/Vα domains and VH/Vβ domains respectively. The CDR3s in both VH and Vβ have a wide range of lengths. Their length distributions are similar and are both centred on a length of 12 residues. The CDR3s of VL and Vα have very different length distributions. The antibody loop (VL CDR3) is almost always 9 residues long whilst CDR3 in the TCR domain (Vα) is longer with a modal length of 13 residues. The VL/Vα CDR3 loop is partially packed in the domain-domain interface. The fact that Vα CDR3 is generally longer than VL CDR3 can be related to the observed difference in the HC2 twist angle (Figure S4 in Text S1) between TCRs and antibodies. The location of the loop at the interface means that in order to compensate for a larger number of residues TCRs tend to reduce the HC2 angle as the interface twists open.

To test the effect of the VL CDR3 length on orientation in antibodies we identified all non-redundant structures in SAbDab with CDRL3 loops of 13 residues. Nine such antibodies exist (Table S3 in Text S1). Eight of them have orientations that are typical of antibodies. This would suggest that having a long CDRL3 loop alone does not promote a TCR-like orientation. One antibody, 3B5H10 Fab, (PDB structures 3s96 and 4dcq [52]) has an orientation similar to that of the TCRs. However, four of the other antibodies in this set have the same light variable germline subgroup (mouse IGLV3), are at least 95% identical sequences, and have identical L3 loops to 3B5H10. They are paired with different heavy chains suggesting that it is VH interface residues that give rise to the difference in orientation.

To isolate these positions, we compared the VH framework residues involved in contacts in the five IGLV3 structures (Table S4 in Text S1). Fifteen VH positions are involved in contacts in all the structures. In one position, IMGT 50, the antibody with the TCR-like orientation is a phenylalanine whilst the other four have leucine at the same position. This large aromatic residue makes contact with the CDRL3 loop and is packed in the periphery of the interface. There are nine other structures in SAbDab with non-identical sequences that have phenylalanine at the same position. In each case, the residue has been mutated from the germline leucine. Seven of these have small HC2 angles (<115°) similar to that observed in TCRs. This suggests that making a mutation from leucine to phenylalanine at IMGT position H50 pushes the VH-VL orientation towards a more TCR-like conformation.

IMGT position 50 in Vβ domains is also predominantly leucine. In TCRs the orientation therefore appears to be achieved using other interface positions. To examine how a VH-VL interface could be made more TCR-like, we examined the relative amino-acid frequencies at interface positions. Framework positions were identified that are conserved in both TCRs and in antibodies (>50%) but with a different amino acid in each set. Ten positions were identified and are listed in Table 2. In order for a position to provide a feasible target for engineering it is preferable for a potential mutation to have been observed naturally. We therefore consulted the Abysis database [53] to find those positions where the conserved TCR amino-acid is also observed at the same position in 2% or more of non-identical antibody sequences. We find five such positions and refer to them as antibody compatible mutations.

**Table 2:** Interface positions that are conserved in both TCRs and antibodies but with a different amino acid.

Position	Antibody Residue (%)	TCR Residue (%)	Antibody Compatible Mutation?
H/β 103	Y (80)	F (62)	Yes
H/β 100	A (93)	S (56)	No
H/β 120	Q (76)	P (51)	Yes
H/β 52	W (95)	L (72)	No
H/β 72	K (78)	P (69)	No
H/β 118	W (90)	F (97)	No
H/β 6	E (53)	Q (97)	Yes
H/β 4	L (98)	V (79)	Yes
H/β 42	V (75)	Y (97)	No
L/α 4	M (55)	V (72)	Yes

Each of the antibody compatible mutations is located on the periphery of the VH-VL interface. Positions from a similar region of the domain interface were previously found to be influential for variation in the HC2 measure of VH-VL orientation [16]. Examining the sequences of the TCR-like antibodies finds that only the antibody that binds in the TCR canonical binding mode and has the most TCR-like variable domain orientation (3cvh) has the conserved TCR amino acid at any of these five positions (glutamine at H/β 6). When the ABangle measures for antibodies with the TCR amino-acid at these positions are calculated we find that two of the four positions, H6 and H4, select for structures with small HC2 angles indicative of the TCR-like orientations.

In summary, the packing of the CDR3 of Vα and VL domains is likely to be an influential factor for domain orientation. However, having a long antibody CDRL3 does not necessarily replicate the TCR orientation. Instead, the VH-VL orientation can be made more TCR-like by adopting specific amino acids at certain interface positions: phe-H50, gln-H6 and val-H4.

## Discussion

The orientation between the antibody variable domains, VH and VL, has previously been found to be influential in determining the geometry of the antigen binding site [14]–[16]. Here, we have compared the VH-VL orientation to the analogous property in the T-Cell receptor, the Vβ-Vα orientation. The two receptor types have distinct sets of orientations. Using the ABangle methodology [16], we characterised how the orientation differs. The best descriptor of orientation difference is the change in the HC2 bend angle. This corresponds to a twisting-like change of the VH or Vβ domain with respect to the VL and Vα domains respectively.

The functional implications of variable domain orientation were investigated by analysing its effect on the structure of a TCR-MHC complex. The Vβ-Vα orientation was changed to assume the orientation of a set of antibody structures and a set of other TCR structures. The contacts, clashes and energetics of the transformed complex structures were analysed. Antibody orientations are found to be incompatible with binding to the MHC in a canonical manner. In contrast, assuming orientations of other TCRs disrupted the complex structure far less. The TCR and MHC are thought to have co-evolved over millions of years allowing for their interaction to be optimised [54]. Variable domain orientation may be one example of a structural property that the TCR has evolved to enhance its ability to recognise the MHC. Alternatively, such properties may not be encoded on the genome but instead selected for during thymic education of T-cells [55]. Therefore, wider ranges of Vβ-Vα orientations may be possible but only particular conformations are chosen as they are structurally compatible with MHC and co-receptor interactions [56].

Despite the apparent steric restriction imposed by their variable domain orientations, it is possible to engineer antibodies that bind to the MHC. The small number of structures of TCR-like antibodies bound to an MHC were examined and their docking angle calculated. Only one of the three cases, 3cvh, binds in the canonical TCR binding mode. This antibody has a VH-VL orientation that is similar to the TCRs. The other TCR-like antibodies also bind in a diagonal mode but use their VH domains in the same way as a TCR uses its Vα domain. Reversing the equivalence of the domains allows them to overcome the steric restrictions imposed by the variable domain orientation. The current number of available structures is small so it is not possible to make statistically robust conclusions. As more structures become available how TCR-like antibodies bind the MHC might be better understood. To understand how an antibody may be engineered to bind specifically to the MHC in a canonical manner, we examined the factors that cause antibodies and TCRs to be distinct in the HC2 angle.

TCRs tend to have longer Vα CDR3 loops than antibodies have VL CDR3 loops. The Vα CDR3 packs into the domain-domain interface and acts to open up the Vβ-Vα orientation. However, antibody structures with similar longer VL CDR3 loops are generally not observed to share the TCR orientation. The length of the loop alone is therefore not predictive of the variable domain orientation. However, a TCR-like orientation is found in antibodies with particular interface residues. The most influential position we identified was at IMGT position 50 on the VH domain. Here, antibodies with a phenylalanine instead of the germline leucine tend to have a TCR-like orientation. The TCR itself predominantly has a leucine at this position. Thus in TCRs the interface is twisted open due to packing a longer Vα CDR3 in the interface but in antibodies the same effect is achieved by the incorporation of a bulky residue, phenylalanine, in the interface. Along with IMGT 50 on the VH domain, additional candidate positions were also identified that could be mutated to increase the similarity of the VH-VL interface to the Vβ-Vα interface. Together, these positions provide promising targets for the rational engineering of antibodies specific to MHCs.

## Supporting Information

Text S1 All supplementary figures and tables are contained within this file.
(DOCX)

## References

[1] Halaby DM, Poupon A, Mornon J (1999) The immunoglobulin fold family: sequence analysis and 3D structure comparisons. Protein Eng 12: 563–571. 10436082 10.1093/protein/12.7.563

[2] Chothia C, Lesk AM (1987) Canonical Structures for the Hypervariable Regions of Immunoglobulins. J Mol Biol 196: 901–917. 3681981 10.1016/0022-2836(87)90412-8

[3] Wu T, Kabat E (1970) An analysis of the sequences of the variable regions of Bence Jones proteins and myeloma light chains and their implications for antibody complementarity. J Exp Med 132: 211–250. 5508247 10.1084/jem.132.2.211 PMC2138737

[4] Abhinandan KR, Martin ACR (2008) Analysis and improvements to Kabat and structurally correct numbering of antibody variable domains. Mol Immunol 45: 3832–3839. 18614234 10.1016/j.molimm.2008.05.022

[5] Pullen AM, Wade T, Marrack P, Kappler JW (1990) Identification of the region of T cell receptor beta chain that interacts with the self-superantigen MIs-1a. Cell 61: 1365–1374. 1694725 10.1016/0092-8674(90)90700-o

[6] Klein MH, Concannon P, Everett M, Kim LD, Hunkapiller T, et al (1987) Diversity and structure of human T-cell receptor alpha-chain variable region genes. Proc Natl Acad Sci U S A 84: 6884–6888. 3502713 10.1073/pnas.84.19.6884 PMC299189

[7] Kuroda D, Shirai H, Jacobson MP, Nakamura H (2012) Computer-aided antibody design. Protein Eng Des Sel 25: 507–522. 22661385 10.1093/protein/gzs024 PMC3449398

[8] Chothia C, Novotný J, Bruccoleri R, Karplus M (1985) Domain Association in Immunoglobulin: The Packing of Variable Domains. J Mol Biol 186: 651–663. 4093982 10.1016/0022-2836(85)90137-8

[9] Vargas-Madrazo E, Paz-García E (2003) An improved model of association for VH-VL immunoglobulin domains: asymmetries between VH and VL in the packing of some interface residues. J Mol Rec 16: 113–120. 10.1002/jmr.613 12833565

[10] Banfield MJ, King DJ, Mountain A, Brady RL (1997) VL:VH domain rotations in engineered antibodies: crystal structures of the Fab fragments from two murine antitumor antibodies and their engineered human constructs. Proteins 29: 161–171. 9329081 10.1002/(sici)1097-0134(199710)29:2<161::aid-prot4>3.0.co;2-g

[11] Teplyakov A, Obmolova G, Malia T, Gilliland G (2011) Antigen recognition by antibody C836 through adjustment of V L/V H packing. Acta Crystallogr Sect F 67: 1165–1167. 10.1107/S1744309111027746 PMC3212354 22102019

[12] Stanfield R, Takimoto-Kamimura M, Rini J, Profy AT, Wilson IA (1993) Major antigen-induced domain rearrangements in an antibody. Structure 1: 83–93. 8069628 10.1016/0969-2126(93)90024-b

[13] Almagro JC, Hernandez-Guzman F, Maier J, Shaulsky J, Butenhof K, et al (2011) Antibody modeling assessment. Proteins 79: 3050–3066. 21935986 10.1002/prot.23130

[14] Abhinandan KR, Martin ACR (2010) Analysis and prediction of VH/VL packing in antibodies. Protein Eng Des Sel 23: 689–697. 20591902 10.1093/protein/gzq043

[15] Chailyan A, Marcatili P, Tramontano A (2011) The association of heavy and light chain variable domains in antibodies: implications for antigen specificity. FEBS 278: 2858–2866. 10.1111/j.1742-4658.2011.08207.x PMC3562479 21651726

[16] Dunbar J, Fuchs A, Shi J, Deane CM (2013) ABangle: characterising the VH-VL orientation in antibodies. Protein Eng Des Sel 24: 611–620. 23708320 10.1093/protein/gzt020

[17] Sivasubramanian A, Sircar A, Chaudhury S, Gray JJ (2009) Toward high-resolution homology modeling of antibody Fv regions and application to antibody-antigen docking. Proteins 74: 497–514. 19062174 10.1002/prot.22309 PMC2909601

[18] Rudolph MG, Stanfield RL, Wilson IA (2006) How TCRs bind MHCs, peptides, and coreceptors. Annu Rev Immunol 24: 419–466. 16551255 10.1146/annurev.immunol.23.021704.115658

[19] Rammensee HG (1995) Chemistry of peptides associated with MHC class I and class II molecules. Curr Opin Immunol 7: 85–96. 7772286 10.1016/0952-7915(95)80033-6

[20] Rammensee H, Bachmann J, Emmerich NP, Bachor Oa, Stevanović S (1999) SYFPEITHI: database for MHC ligands and peptide motifs. Immunogenetics 50: 213–219. 10602881 10.1007/s002510050595

[21] Robinson J, Halliwell JA, McWilliam H, Lopez R, Parham P, et al (2013) The IMGT/HLA database. Nucleic Acids Res 41: D1222–1227. 23080122 10.1093/nar/gks949 PMC3531221

[22] Rudolph MG, Luz JG, Wilson IA (2002) Structural and thermodynamic correlates of T cell signaling. Annu Rev Biophys Biomol Struct 31: 121–149. 11988465 10.1146/annurev.biophys.31.082901.134423

[23] Yin Y, Li Y, Mariuzza Ra (2012) Structural basis for self-recognition by autoimmune T-cell receptors. Immunol Rev 250: 32–48. 23046121 10.1111/imr.12002

[24] Wucherpfennig K, Call M (2009) Structural alterations in peptide–MHC recognition by self-reactive T cell receptors. Curr Opin Immunol 21: 590–595. 19699075 10.1016/j.coi.2009.07.008 PMC2787854

[25] Dahan R, Reiter Y (2012) T-cell-receptor-like antibodies - generation, function and applications. Exp Rev Mol Med 14: e6. 10.1017/erm.2012.2 22361332

[26] Cohen M, Reiter Y (2013) T-Cell Receptor-Like Antibodies: Targeting the Intracellular Proteome Therapeutic Potential and Clinical Applications. Antibodies 2: 517–534.

[27] Molloy PE, Sewell AK, Jakobsen BK (2005) Soluble T cell receptors: novel immunotherapies. Curr Opin Pharmacol 5: 438–443. 15939669 10.1016/j.coph.2005.02.004

[28] Neumann F, Sturm C, Hülsmeyer M, Dauth N, Guillaume P, et al (2009) Fab antibodies capable of blocking T cells by competitive binding have the identical specificity but a higher affinity to the MHC-peptide-complex than the T cell receptor. Immunology letters 125: 86–92. 19524620 10.1016/j.imlet.2009.06.002

[29] Dostalek M, Gardner I, Gurbaxani BM, Rose RH, Chetty M (2013) Pharmacokinetics, pharmacodynamics and physiologically-based pharmacokinetic modelling of monoclonal antibodies. Clin Pharmacokinet 52: 83–124. 23299465 10.1007/s40262-012-0027-4

[30] Andersen PS, Stryhn a, Hansen BE, Fugger L, Engberg J, et al (1996) A recombinant antibody with the antigen-specific, major histocompatibility complex-restricted specificity of T cells. Proc Natl Acad Sci U S A 93: 1820–1824. 8700842 10.1073/pnas.93.5.1820 PMC39865

[31] Cohen CJ, Denkberg G, Lev A, Epel M, Reiter Y (2003) Recombinant antibodies with MHC-restricted, new tools to study antigen presentation and TCR- peptide-MHC interactions. J Mol Recognit 16: 324–332. 14523945 10.1002/jmr.640

[32] Biddison WE, Turner RV, Gagnon SJ, Lev a, Cohen CJ, et al (2003) Tax and M1 Peptide/HLA-A2-Specific Fabs and T Cell Receptors Recognize Nonidentical Structural Features on Peptide/HLA-A2 Complexes. J Immunol 171: 3064–3074. 12960332 10.4049/jimmunol.171.6.3064

[33] Stewart-Jones G, Wadle A, Hombach A, Shenderov E, Held G, et al (2009) Rational development of high-affinity T-cell receptor-like antibodies. Proceedings of the National Academy of Sciences of the United States of America 106: 5784–5788. 19307587 10.1073/pnas.0901425106 PMC2667008

[34] Hülsmeyer M, Chames P, Hillig RC, Stanfield RL, Held G, et al (2005) A major histocompatibility complex-peptide-restricted antibody and t cell receptor molecules recognize their target by distinct binding modes: crystal structure of human leukocyte antigen (HLA)-A1-MAGE-A1 in complex with FAB-HYB3. J Biol Chem 280: 2972–2980. 15537658 10.1074/jbc.M411323200

[35] Mareeva T, Lebedeva T, Anikeeva N, Manser T, Sykulev Y (2004) Antibody specific for the peptide.major histocompatibility complex. Is it T cell receptor-like? J Biol Chem 279: 44243–44249. 15302863 10.1074/jbc.M407021200

[36] McBeth C, Seamons A, Pizarro JC, Fleishman SJ, Baker D, et al (2008) A New Twist In TCR Diversity Revealed By A Forbidden αβ TCR. J Mol Biol 375: 1306–1319. 18155234 10.1016/j.jmb.2007.11.020 PMC2330282

[37] Berman HM, Westbrook J, Feng Z, Gilliland G, Bhat TN, et al (2000) The Protein Data Bank. Nucleic Acids Res 28: 235–242. 10592235 10.1093/nar/28.1.235 PMC102472

[38] Lefranc M-P, Giudicelli V, Ginestoux C, Jabado-Michaloud J, Folch G, et al (2009) IMGT, the international ImMunoGeneTics information system. Nucleic Acids Res 37: D1006–1012. 18978023 10.1093/nar/gkn838 PMC2686541

[39] Li W, Godzik A (2006) Cd-hit: a fast program for clustering and comparing large sets of protein or nucleotide sequences. Bioinformatics 22: 1658–1659. 16731699 10.1093/bioinformatics/btl158

[40] Dunbar J, Krawczyk K, Leem J, Baker T, Fuchs A, et al (2014) SAbDab: the structural antibody database. Nucleic Acids Res 42: D1140–D1146. 24214988 10.1093/nar/gkt1043 PMC3965125

[41] Ehrenmann F, Kaas Q, Lefranc M-P (2010) IMGT/3Dstructure-DB and IMGT/DomainGapAlign: a database and a tool for immunoglobulins or antibodies, T cell receptors, MHC, IgSF and MhcSF. Nucleic Acids Res 38: D301–307. 19900967 10.1093/nar/gkp946 PMC2808948

[42] Altschul SF, Gish W, Miller W, Myers EW, Lipman DJ (1990) Basic local alignment search tool. J Mol Biol 215: 403–410. 2231712 10.1016/S0022-2836(05)80360-2

[43] Cock PJA, Antao T, Chang JT, Chapman BA, Cox CJ, et al (2009) Biopython: freely available Python tools for computational molecular biology and bioinformatics. Bioinformatics 25: 1422–1423. 19304878 10.1093/bioinformatics/btp163 PMC2682512

[44] Maechler M, Rousseeuw P, Struyf A, Hubert M, Hornik K (2013) cluster: Cluster Analysis Basics and Extensions.

[45] Knapp B, Dunbar J, Deane CM (2014) Large Scale Characterization of the LC13 TCR and HLA-B8 Structural Landscape in Reaction to 172 Altered Peptide Ligands: A Molecular Dynamics Simulation Study. Plos Comp Bio In Press. 10.1371/journal.pcbi.1003748 PMC4125040 25101830

[46] Mareeva T, Martinez-Hackert E, Sykulev Y (2008) How a T cell receptor-like antibody recognizes major histocompatibility complex-bound peptide. J Biol Chem 283: 29053–29059. 18703505 10.1074/jbc.M804996200 PMC2570882

[47] Hennecke J, Wiley DC (2001) T cell receptor-MHC interactions up close. Cell 104: 1–4. 11163234 10.1016/s0092-8674(01)00185-4

[48] Kjer-Nielsen L, Clements CS, Purcell AW, Brooks AG, Whisstock JC, et al (2003) A structural basis for the selection of dominant alphabeta T cell receptors in antiviral immunity. Immunity 18: 53–64. 12530975 10.1016/s1074-7613(02)00513-7

[49] Krivov GG, Shapovalov MV, Dunbrack RL (2009) Improved prediction of protein side-chain conformations with SCWRL4. Proteins 77: 778–795. 19603484 10.1002/prot.22488 PMC2885146

[50] Davis IW, Leaver-Fay A, Chen VB, Block JN, Kapral GJ, et al (2007) MolProbity: all-atom contacts and structure validation for proteins and nucleic acids. Nucleic Acids Res 35: W375–383. 17452350 10.1093/nar/gkm216 PMC1933162

[51] Shen M-Y, Sali A (2006) Statistical potential for assessment and prediction of protein structures. Protein Sci 15: 2507–2524. 17075131 10.1110/ps.062416606 PMC2242414

[52] Peters-Libeu C, Miller J, Rutenber E, Newhouse Y, Krishnan P, et al (2012) Disease-associated polyglutamine stretches in monomeric huntingtin adopt a compact structure. J Mol Biol 421: 587–600. 22306738 10.1016/j.jmb.2012.01.034 PMC3358578

[53] Martin ACR (2010) Antibody Engineering Vol. 2. In: Kontermann R, Dübel S, editors. Antibody Engineering. 2 ed. Berlin, Heidelberg: Springer Berlin Heidelberg. pp. 33–51.

[54] Garcia KC, Adams EJ (2005) How the T cell receptor sees antigen–a structural view. Cell 122: 333–336. 16096054 10.1016/j.cell.2005.07.015

[55] Collins EJ, Riddle DS (2008) TCR-MHC docking orientation: natural selection, or thymic selection? Immunologic research 41: 267–294. 18726714 10.1007/s12026-008-8040-2

[56] Rangarajan S, Mariuzza Ra (2014) T cell receptor bias for MHC: co-evolution or co-receptors? Cell Mol Life Sci 71: 3059–3068. 24633202 10.1007/s00018-014-1600-9 PMC11113676

