# Supplementary material for: Examining Variable Domain Orientations in Antigen Receptors Gives Insight into TCR-Like Antibody Design
Source: PLoS Comput Biol. 2014 Sep 18;10(9):e1003852. doi: 10.1371/journal.pcbi.1003852 (PMC4168974; doi:10.1371/journal.pcbi.1003852)
Supplement: Text S1 — All supplementary figures and tables are contained within this file. (DOCX) [file pcbi.1003852.s001.docx]

# Supplementary material

## Supplementary figures


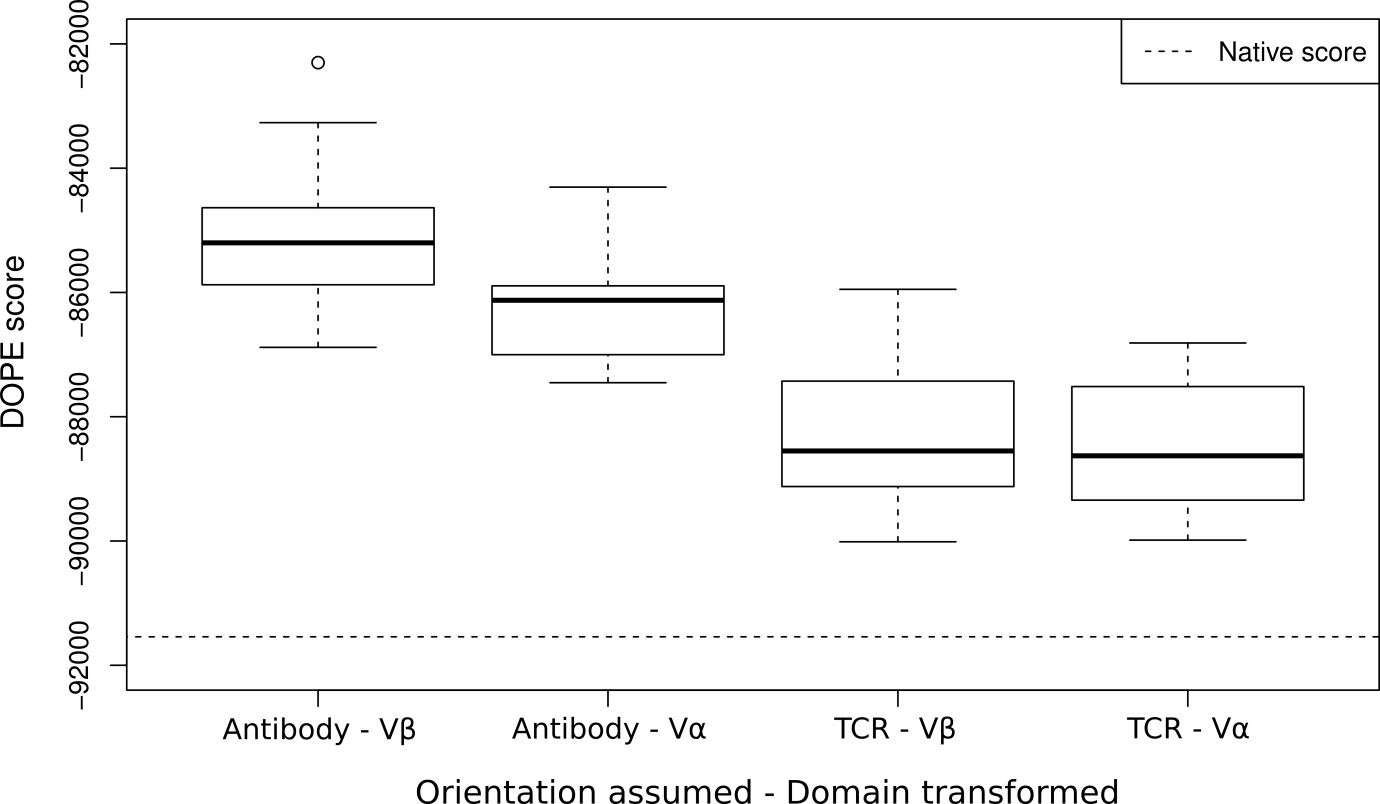


**Figure S1**: The DOPE scores of the TCR/MHC-peptide complex structure when placed in Vβ-Vα orientations assumed from the antibody decoy set and the TCR decoy set. The first boxplot corresponds to keeping the Vα domain in its native position and transforming the Vβ domain to positions observed in antibodies. The second boxplot is analogous to the first except now the Vβ domain is fixed and the Vα domain is repositioned. The third and fourth distributions are the respective boxplots for the TCR decoy set. The DOPE score is significantly higher for the antibody decoys than the TCR decoys representing less favourable interactions for the antibody orientations.


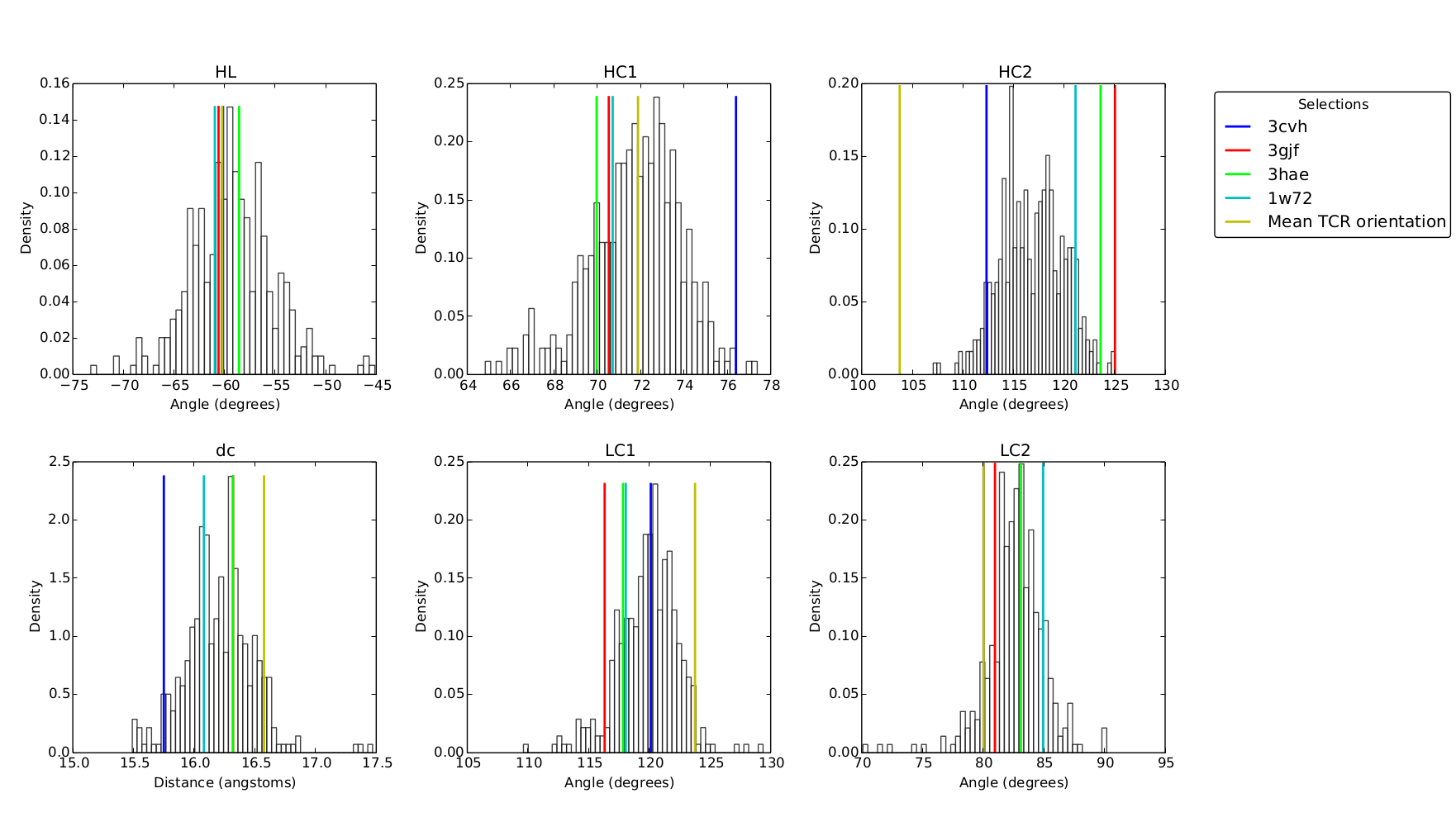


**Figure S2**: Orientation measures of TCR-like antibodies and the mean orientation of TCRs compared to the distribution of general antibody orientations (histograms). The yellow vertical line is the position of the mean orientation for TCRs. Each remaining vertical line shows the location of the orientation measures for a single pair of variable domains of a TCR-like antibody. The difference in orientation between TCRs and antibodies is best described using the HC2 twist angle. Only one antibody, 3cvh, has a TCR-like orientation in this measure.


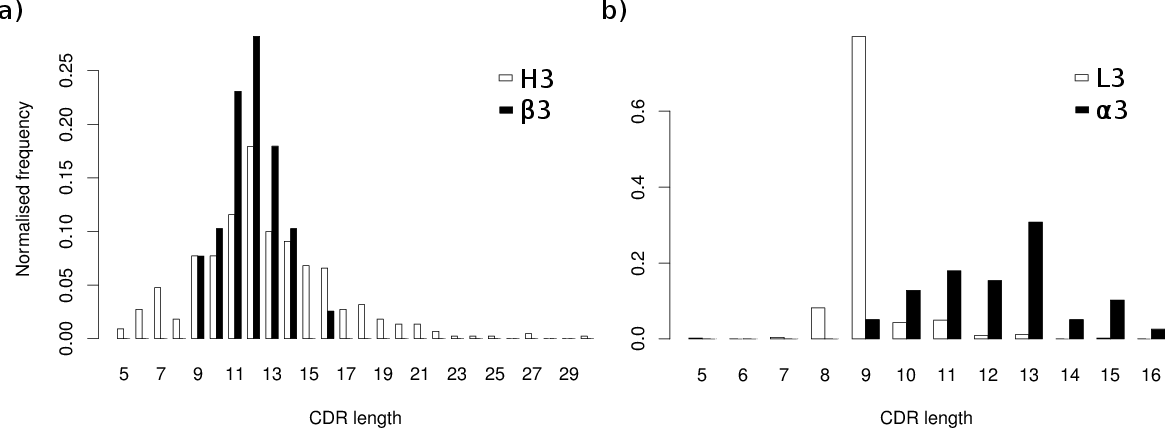


**Figure S3**: Length distributions of the CDR3 in a) the equivalent VH and Vβ domains and b) VL and equivalent Vα domains. In each case the IMGT definition of CDR has been used.


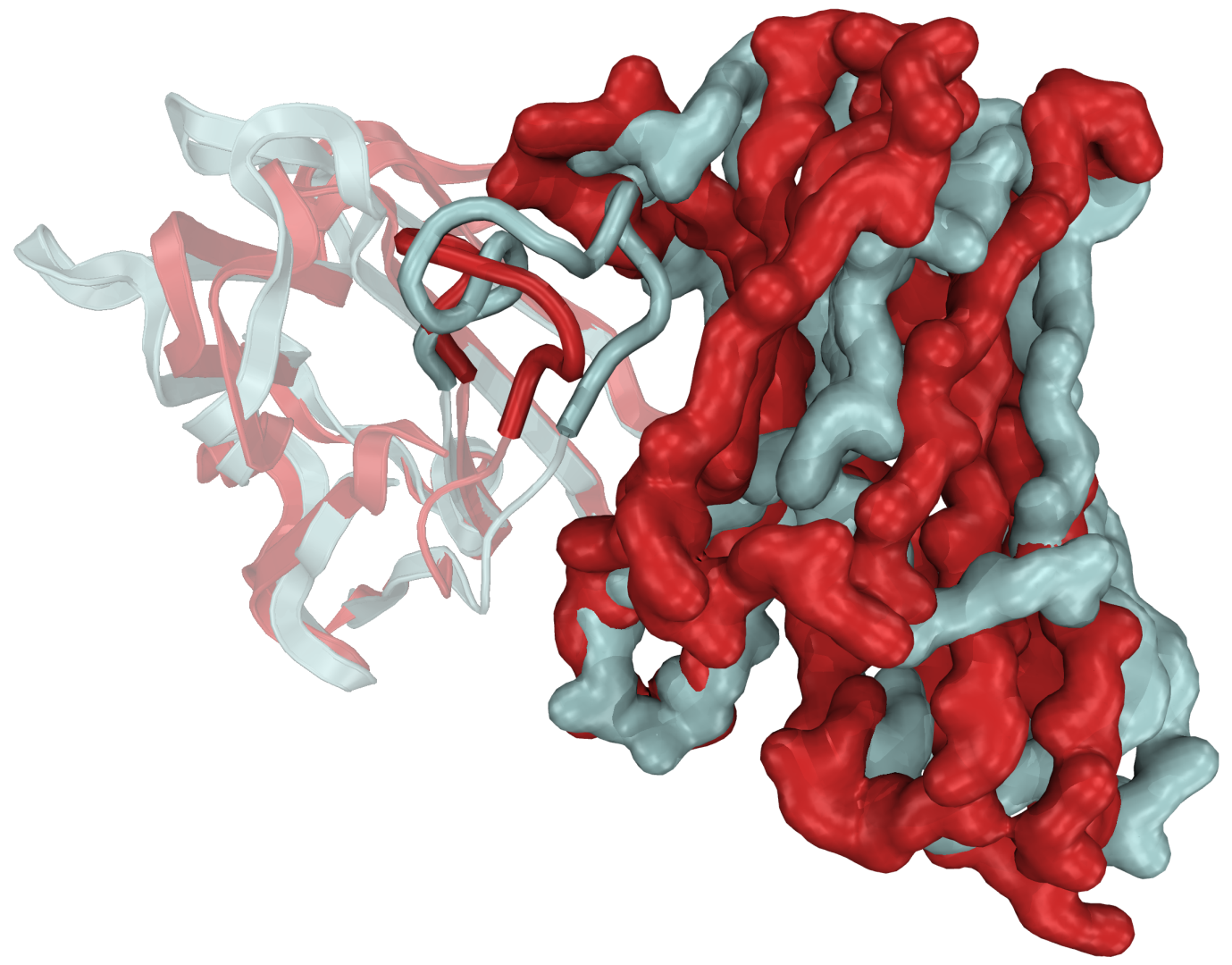


**Figure S4**: The influence of the VL/Vα CDR3 on variable domain orientation. A representative TCR is shown in cyan and an antibody in red. The structures are aligned using the shared framework positions of the VL and Vα domains (left-ribbon). On the right are the VH and Vβ domains. The Vβ domain twists relative to the position that the VH domain sits in. The difference in orientation can be explained by the different packing of the CDRL3 and the longer CDRα3 loops (shown in ribbon representation). The TCR loop packs into the domain interface and opens up the orientation whilst the shorter antibody loop makes fewer inter-chain contacts. The difference in orientation is captured in our HC2 angle measure.


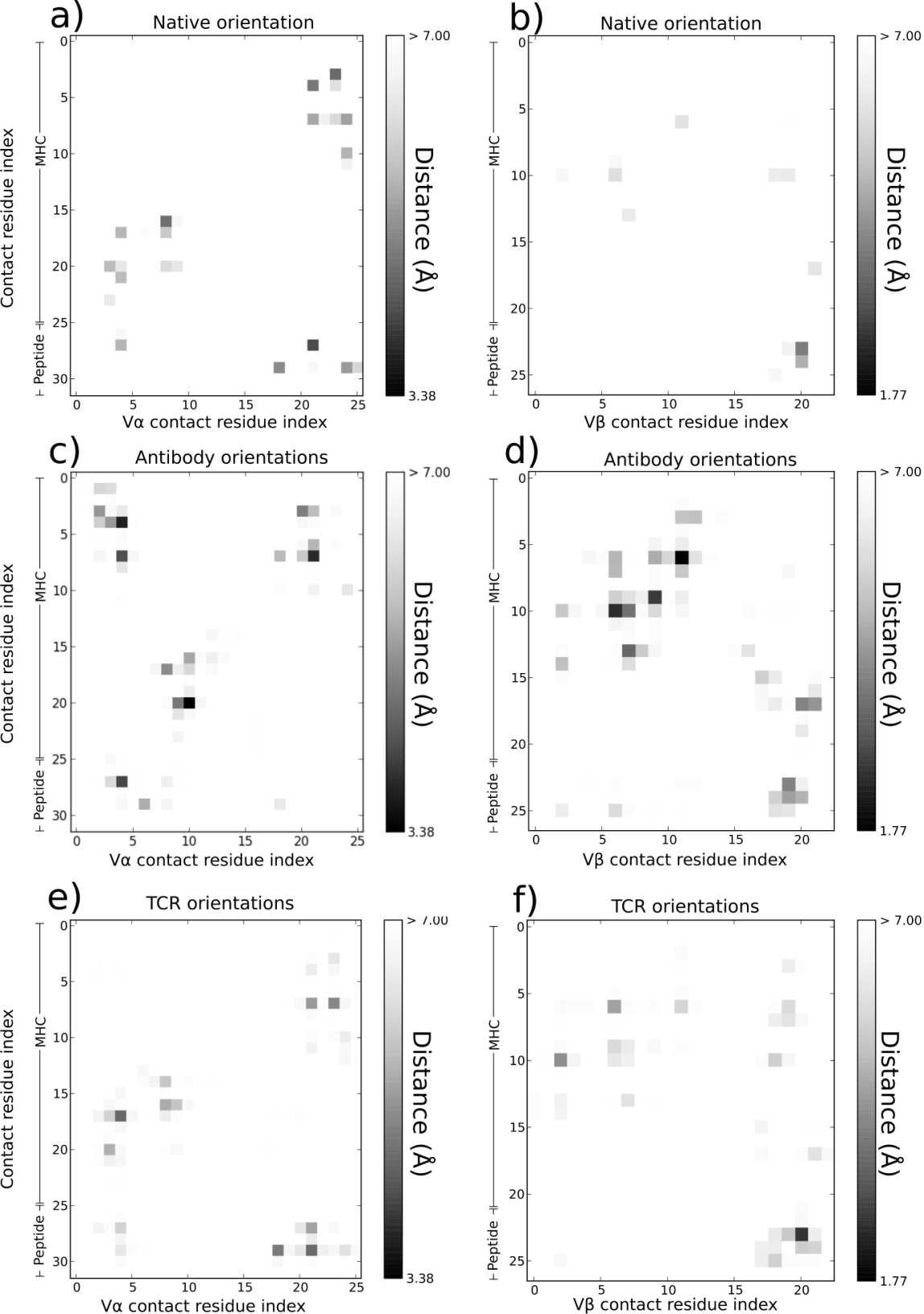


**Figure S5**: a) The Cβ-Cβ contact distances between the Vα domain and the MHC/peptide in the native complex. b) The corresponding image for Vβ and the MHC/peptide contacts. The TCR is made to assume variable orientations taken from the antibody decoy set. The mean contact distance over all of these decoys for c) the Vα domain and d) the Vβ domain is calculated. Similarly, the mean contact distances for e) the Vα domain and f) the Vβ domain are calculated when the structure assumes orientations from other TCR. When antibody orientations are assumed, unfavourable clashes are induced between the TCR and MHC. When other TCR orientations are assumed, the complex is disrupted to a lesser extent.

##
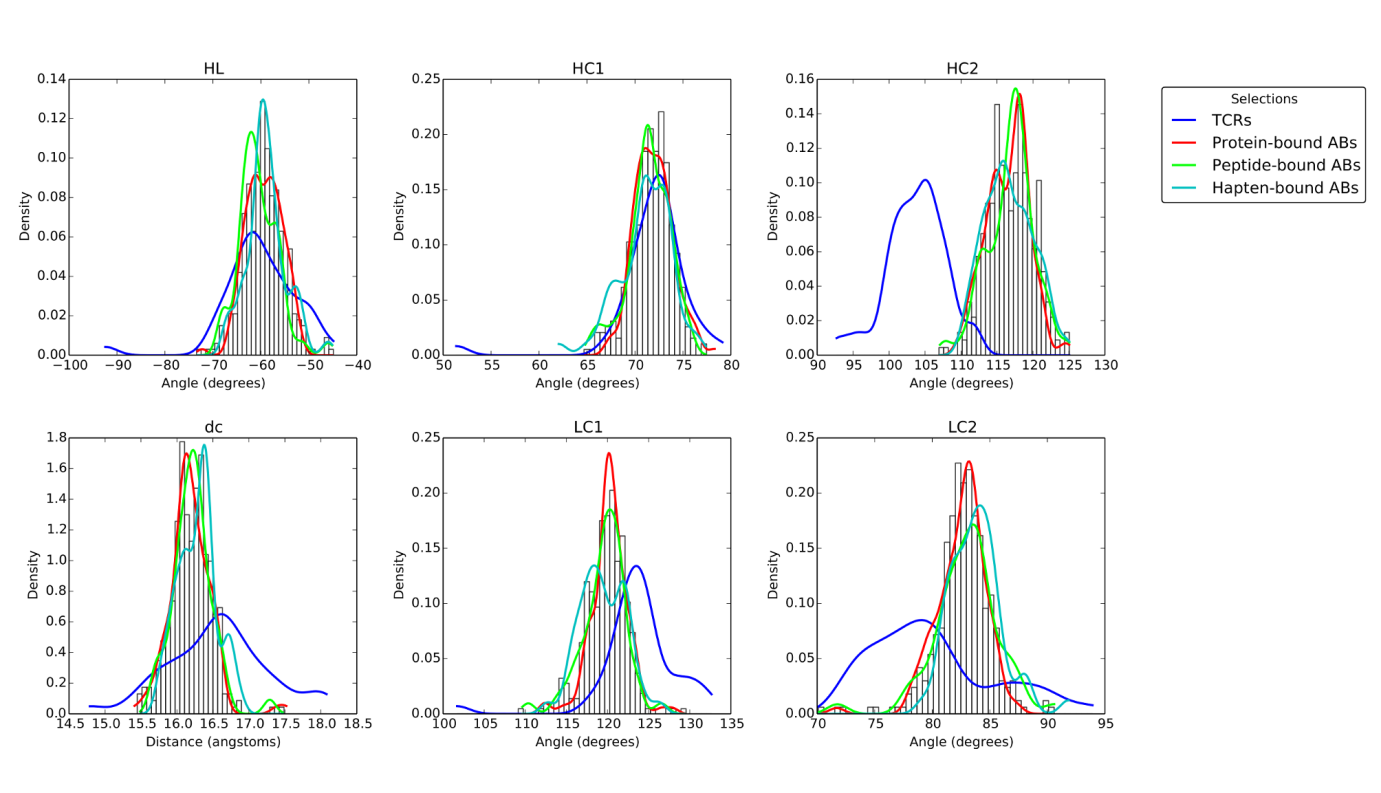


**Figure S6:** The distributions of ABangle orientation measures for TCRs and antibodies stratified by the type of antigen they are bound to (Protein, Peptide or Hapten). The histograms show the background distribution for general antibodies. No preference in orientation is found for any of the three antibody groups. Thus, the antigen type is found not to determine the absolute VH-VL orientation. No group is any more similar to the TCR distribution than to the general antibody distribution.

## Supplementary Tables

**Table S1**: The antibody decoy set

| PDB | Heavy Chain | Light Chain |
| --- | --- | --- |
| 3o11 | H | L |
| 1kb9 | J | K |
| 3qeg | H | L |
| 3qos | B | A |
| 43c9 | F | E |
| 3o2d | H | L |
| 1iqw | H | L |
| 1fpt | H | L |
| 1ind | H | L |
| 3eyq | D | C |
| 1rjl | B | A |
| 3h42 | H | L |
| 2e27 | H | L |
| 1sy6 | H | L |
| 1rur | H | L |
| 3c08 | H | L |
| 1yee | H | L |
| 3sob | H | L |
| 1kel | H | L |
| 3nh7 | H | L |

**Table S2**: The TCR decoy set

| PDB | Alpha Chain | Beta Chain |
| --- | --- | --- |
| 3utt | I | J |
| 3ffc | D | E |
| 3kpr | I | J |
| 1ymm | D | E |
| 1nfd | A | B |
| 3dx9 | A | B |
| 2ial | A | B |
| 3qiw | C | D |
| 3pl6 | C | D |
| 3mff | A | B |
| 2esv | D | E |
| 3rgv | A | B |
| 3qib | C | D |
| 1fo0 | A | B |
| 2uwe | L | M |
| 2xna | A | B |
| 3he6 | C | D |
| 3he7 | C | D |
| 3o6f | C | D |
| 3rev | A | B |

**Table S3**: Antibodies with length 13 CDR L3 loops

| PDB code |
| --- |
| 3mlw |
| 2qhr |
| 4isv |
| 2b1h |
| 3ffd |
| 3s96 |
| 2otu |
| 4ht1 |
| 4jo2 |

**Table S4**: Heavy contact positions in L3 length 13 structures with IGLV3 subgroup

| Structure | 42 | 44 | 48 | 49 | 50 | 51 | 52 | 55 | 66 | 98 | 103 | 118 | 119 | 120 | 121 |
| --- | --- | --- | --- | --- | --- | --- | --- | --- | --- | --- | --- | --- | --- | --- | --- |
| 2qhr H L | V | Q | K | R | L | E | W | Y | Y | P | Y | W | G | Q | G |
| 4isv B A | V | Q | K | G | L | K | W | W | T | A | F | W | G | Q | G |
| 3ffd A B | I | Q | K | R | L | E | W | T | Y | P | Y | W | G | Q | G |
| 2otu D C | V | Q | K | R | L | E | W | F | Y | P | Y | W | G | Q | G |
| 3s96 A B | V | Q | K | G | **F** | E | W | W | T | V | F | W | G | Q | G |
| TCRs  Modal % | Y 100 | Q 97 | H 25 | G  77 | L 82 | R 49 | L 74 | Y 64 | T 56 | Q 59 | F 64 | F 100 | G 100 | P 51 | G 100 |

**Table S5**: IMGT positions that make up the Vα and Vβ coresets

| Vα coreset | Vβ coreset |
| --- | --- |
| 16 | 5 |
| 17 | 6 |
| 18 | 8 |
| 20 | 9 |
| 22 | 10 |
| 38 | 11 |
| 39 | 12 |
| 40 | 14 |
| 41 | 15 |
| 42 | 16 |
| 43 | 22 |
| 44 | 23 |
| 45 | 24 |
| 52 | 38 |
| 85 | 39 |
| 86 | 40 |
| 88 | 41 |
| 89 | 42 |
| 90 | 43 |
| 94 | 44 |
| 95 | 51 |
| 96 | 52 |
| 97 | 53 |
| 98 | 86 |
| 99 | 99 |
| 100 | 100 |
| 101 | 101 |
| 102 | 102 |
| 103 | 103 |
| 104 | 104 |

**Table S6: The non-redundant antibody dataset**

| PDB | Heavy Chain | Light Chain |
| --- | --- | --- |
| 3hnv | H | L |
| 3lrs | A | B |
| 1um4 | H | L |
| 1ct8 | D | C |
| 4etq | A | B |
| 3ls4 | H | L |
| 1k6q | H | L |
| 1eap | B | A |
| 2yc1 | A | B |
| 4dvb | A | B |
| 1yy9 | D | C |
| 2cmr | H | L |
| 1nca | H | L |
| 15c8 | H | L |
| 2r29 | H | L |
| 3qwo | A | B |
| 3kym | B | A |
| 3hi6 | H | L |
| 2gfb | L | K |
| 2adg | B | A |
| 3giz | H | L |
| 3g6a | B | A |
| 2vl5 | C | D |
| 1fn4 | B | A |
| 2z4q | B | A |
| 2r8s | H | L |
| 2bjm | H | L |
| 2gcy | B | A |
| 3ma9 | H | L |
| 1ibg | H | L |
| 2aj3 | B | A |
| 1ob1 | E | D |
| 3bgf | B | C |
| 1jrh | H | L |
| 2osl | A | B |
| 1iai | H | L |
| 1xiw | D | C |
| 1clo | H | L |
| 3mnz | B | A |
| 12e8 | H | L |
| 1fh5 | H | L |
| 1dfb | H | L |
| 1opg | H | L |
| 1gig | H | L |
| 3phq | B | A |
| 1rih | H | L |
| 3hi1 | B | A |
| 3o2v | H | L |
| 2xqb | H | L |
| 3m8o | H | L |
| 1kfa | H | L |
| 1sbs | H | L |
| 4fql | H | L |
| 3ifo | H | L |
| 1rzi | N | M |
| 2hrp | H | L |
| 1yee | H | L |
| 4fqj | H | L |
| 4ers | H | L |
| 1etz | H | L |
| 3bn9 | F | E |
| 1i3g | H | L |
| 2uzi | H | L |
| 1dl7 | H | L |
| 2eiz | B | A |
| 7fab | H | L |
| 1hh9 | B | A |
| 1psk | H | L |
| 1c5c | H | L |
| 2vxt | H | L |
| 2z92 | A | B |
| 1tqb | B | C |
| 1bgx | H | L |
| 1pg7 | I | M |
| 2hh0 | H | L |
| 1iqd | B | A |
| 2atk | A | B |
| 3qpx | H | L |
| 3dif | B | A |
| 3ujt | H | L |
| 3liz | H | L |
| 1b2w | H | L |
| 3mlt | H | L |
| 2qsc | H | L |
| 1wz1 | H | L |
| 1a4k | H | L |
| 3ck0 | H | L |
| 1pkq | B | A |
| 1iqw | H | L |
| 3pp3 | I | K |
| 1h0d | B | A |
| 3ra7 | H | L |
| 2jb5 | H | L |
| 1xgy | I | M |
| 3l1o | H | L |
| 3i9g | H | L |
| 3na9 | H | L |
| 1a14 | H | L |
| 2j4w | H | L |
| 1uz8 | H | L |
| 1dee | D | C |
| 1fns | H | L |
| 2xza | H | L |
| 1rzf | H | L |
| 1bj1 | H | L |
| 3fn0 | H | L |
| 1egj | H | L |
| 3i2c | H | L |
| 1keg | H | L |
| 3ojd | B | A |
| 1a3r | H | L |
| 1wt5 | A | C |
| 1igj | B | A |
| 1rmf | H | L |
| 2qqn | H | L |
| 2uyl | N | M |
| 1m7d | B | A |
| 1w72 | H | L |
| 3ldb | C | B |
| 1dba | H | L |
| 1rur | H | L |
| 1ru9 | H | L |
| 3tnn | E | F |
| 1igf | H | L |
| 3mlx | H | L |
| 3lmj | H | L |
| 4hcr | H | L |
| 3gi9 | H | L |
| 2fb4 | H | L |
| 3qeh | G | H |
| 3bae | H | L |
| 3idx | H | L |
| 3h3p | H | L |
| 3skj | H | L |
| 1eo8 | H | L |
| 1a6t | B | A |
| 1c5d | H | L |
| 1uwe | V | U |
| 3nfp | H | L |
| 2xkn | D | C |
| 3t2n | H | L |
| 3ngb | B | C |
| 1n7m | L | H |
| 3r06 | H | L |
| 2fbj | H | L |
| 1wej | H | L |
| 1lk3 | I | M |
| 1e6j | H | L |
| 3g5y | B | A |
| 2d7t | H | L |
| 3s37 | H | L |
| 3dsf | H | L |
| 1nsn | H | L |
| 4ffv | H | L |
| 1kcv | H | L |
| 1kc5 | H | L |
| 1il1 | A | B |
| 3qrg | H | L |
| 3b9k | H | L |
| 3d85 | B | A |
| 1jhk | H | L |
| 1mlc | D | C |
| 1ad9 | B | A |
| 3qot | H | L |
| 2hff | B | A |
| 1it9 | H | L |
| 1cl7 | H | L |
| 2h1p | H | L |
| 3qos | B | A |
| 1cly | H | L |
| 3mod | H | L |
| 2fl5 | F | E |
| 2agj | H | L |
| 1jfq | H | L |
| 2v7n | F | E |
| 3cmo | H | L |
| 3u0t | B | A |
| 2xra | H | L |
| 1tzi | B | A |
| 3uyr | H | L |
| 43c9 | F | E |
| 1t4k | B | A |
| 1axt | H | L |
| 1uwx | H | L |
| 1t66 | H | L |
| 2g2r | H | L |
| 4dgv | H | L |
| 4ffy | H | L |
| 3iu4 | H | L |
| 1bfv | H | L |
| 1qkz | H | L |
| 3s35 | H | L |
| 2xqy | G | L |
| 3kr3 | H | L |
| 1f90 | H | L |
| 2jel | H | L |
| 2w60 | A | B |
| 2vxv | H | L |
| 1j05 | B | A |
| 3hr5 | B | A |
| 2xwt | A | B |
| 1lo4 | H | L |
| 3inu | M | N |
| 2qhr | H | L |
| 3sy0 | B | A |
| 1gc1 | H | L |
| 1rzg | C | D |
| 4eow | H | L |
| 2orb | H | L |
| 3aaz | H | L |
| 3f58 | H | L |
| 2b1a | H | L |
| 2dtm | H | L |
| 2g75 | A | B |
| 1sy6 | H | L |
| 1rz7 | H | L |
| 3cvh | H | L |
| 3fzu | C | D |
| 3gk8 | H | L |
| 2zpk | H | L |
| 1mam | H | L |
| 3sqo | H | L |
| 1ggc | H | L |
| 3eoa | B | A |
| 3gnm | H | L |
| 3eo0 | D | C |
| 2r56 | I | M |
| 1zan | H | L |
| 1za6 | D | C |
| 1seq | H | L |
| 3bky | H | L |
| 2ok0 | H | L |
| 4f2m | A | B |
| 2ap2 | D | C |
| 1frg | H | L |
| 1y0l | F | E |
| 2xa8 | H | L |
| 1cbv | H | L |
| 1riv | H | L |
| 1hil | B | A |
| 3h0t | B | A |
| 2zch | H | L |
| 1ai1 | H | L |
| 3c2a | I | M |
| 3qhf | H | L |
| 1mnu | H | L |
| 2op4 | H | L |
| 4fqi | H | L |
| 1kel | H | L |
| 1ae6 | H | L |
| 3o45 | A | B |
| 3nps | B | C |
| 1mhh | D | C |
| 1mh5 | H | L |
| 2oqj | H | G |
| 2bmk | H | L |
| 1jhl | H | L |
| 1fe8 | H | L |
| 1hkl | H | L |
| 2w9d | H | L |
| 1bvk | E | D |
| 1jv5 | B | A |
| 2zkh | H | L |
| 4i9w | E | D |
| 1baf | H | L |
| 1yqv | H | L |
| 1ztx | H | L |
| 4g5z | H | L |
| 2v7h | B | A |
| 1nj9 | H | L |
| 1mhp | X | Y |
| 3mxv | H | L |
| 2vdk | H | L |
| 1kb5 | H | L |
| 1dvf | D | C |
| 3cfb | B | A |
| 3q6g | H | L |
| 1nld | H | L |
| 1i8k | B | A |
| 3clf | H | L |
| 4hc1 | M | N |
| 1t3f | B | A |
| 1uj3 | B | A |
| 1ors | B | A |
| 2ddq | H | L |
| 1for | H | L |
| 1b4j | H | L |
| 2e27 | H | L |
| 1hq4 | B | A |
| 2bdn | H | L |
| 3hc4 | H | L |
| 3nzh | H | L |
| 2y6s | D | C |
| 4ala | H | L |
| 1cic | B | A |
| 2adf | H | L |
| 1ind | H | L |
| 3fo2 | B | A |
| 3kdm | B | A |
| 1mrd | H | L |
| 3det | E | F |
| 3tnm | A | B |
| 1aqk | H | L |
| 2j6e | H | L |
| 4evn | M | N |
| 1dqd | H | L |
| 1a5f | H | L |
| 1ynl | H | L |
| 2c1o | B | A |
| 1h3p | H | L |
| 3c08 | H | L |
| 1bfo | B | A |
| 3lex | A | B |
| 2vq1 | F | E |
| 1i7z | B | A |
| 1lo0 | Y | X |
| 1f11 | D | C |
| 3klh | D | C |
| 3go1 | H | L |
| 3rkd | H | L |
| 1jgu | H | L |
| 1pg7 | X | W |
| 1i9i | H | L |
| 3mlw | I | M |
| 3oz9 | H | L |
| 1igt | B | A |
| 1nak | H | L |
| 3qg7 | H | L |
| 1ktr | H | L |
| 1uwg | Y | X |
| 2arj | H | L |
| 1fsk | C | B |
| 3o11 | H | L |
| 2vxq | H | L |
| 2pcp | D | C |
| 3nh7 | I | M |
| 1plg | H | L |
| 1nc2 | D | C |
| 1q0x | H | L |
| 3bqu | D | C |
| 1dsf | H | L |
| 3iet | B | A |
| 3l7e | H | L |
| 3l7f | E | D |
| 3cfd | H | L |
| 1rjl | B | A |
| 3qeg | H | L |
| 3ehb | C | D |
| 3c5s | D | C |
| 2fr4 | B | A |
| 1fig | H | L |
| 1cr9 | H | L |
| 1kb9 | J | K |
| 1f4w | H | L |
| 1yjd | H | L |
| 1zea | H | L |
| 4g6k | H | L |
| 1mfe | H | L |
| 1d5b | B | A |
| 8fab | B | A |
| 1c12 | B | A |
| 3e8u | H | L |
| 2eh7 | H | L |
| 3rvu | D | C |
| 1ad0 | D | C |
| 1qfu | H | L |
| 1ikf | H | L |
| 3n9g | H | L |
| 3ffd | A | B |
| 2a9m | H | L |
| 4dtg | H | L |
| 3eyq | D | C |
| 3eyf | B | A |
| 2aab | H | L |
| 3sob | H | L |
| 3mbx | H | L |
| 3g04 | B | A |
| 1s3k | H | L |
| 2otu | D | C |
| 1fvd | D | C |
| 1fgv | H | L |
| 1fpt | H | L |
| 3nz8 | H | L |
| 3ntc | H | L |
| 3i75 | B | A |
| 2gsi | B | A |
| 2q8b | H | L |
| 1dqq | D | C |
| 1ghf | H | L |
| 1e4w | H | L |
| 1jnl | H | L |
| 2oz4 | H | L |
| 1fj1 | D | C |
| 1mex | H | L |
| 1emt | H | L |
| 1mim | H | L |
| 3qpq | J | I |
| 1f3d | H | L |
| 1igc | H | L |
| 1sm3 | H | L |
| 3ks0 | K | J |
| 1l7i | H | L |
| 3hmx | H | L |
| 3i50 | H | L |
| 2aeq | H | L |
| 1osp | H | L |
| 1aif | B | A |
| 3gje | H | L |
| 2vxs | J | N |
| 1fbi | Q | P |
| 1igm | H | L |
| 1ce1 | H | L |
| 32c2 | B | A |
| 3dgg | D | C |
| 1dn0 | B | A |
| 1jnh | H | G |
| 2fat | H | L |
| 2j88 | H | L |
| 3o0r | H | L |
| 3sdy | H | L |
| 2a6i | B | A |
| 1ejo | H | L |
| 2ak1 | H | L |
| 3vg9 | C | B |
| 2brr | Y | X |
| 1t2q | H | L |
| 1nl0 | H | L |
| 1bln | B | A |
| 1p4b | H | L |
| 1a7o | H | L |
| 2g5b | H | G |
| 3mck | H | L |
| 3h42 | H | L |
| 2ai0 | K | O |
| 3o2d | H | L |
| 1mcp | H | L |
| 3qcu | I | M |
| 3ifp | A | B |
| 1n0x | K | M |
| 3eys | H | L |
| 3ghe | H | L |

**Table S7: The non-redundant TCR dataset**

| PDB | Alpha chain | Beta Chain |
| --- | --- | --- |
| 3c5z | A | B |
| 3gsn | A | B |
| 1g6r | A | B |
| 3qeq | D | E |
| 3pl6 | C | D |
| 3kpr | I | J |
| 3qdj | D | E |
| 3qib | C | D |
| 2xna | A | B |
| 3mbe | C | D |
| 1fo0 | A | B |
| 1ymm | D | E |
| 1j8h | D | E |
| 3ffc | D | E |
| 2gj6 | D | E |
| 3utt | I | J |
| 3rev | A | B |
| 2uwe | L | M |
| 2pye | D | E |
| 1zgl | U | V |
| 2ial | A | B |
| 2ak4 | N | P |
| 3he7 | C | D |
| 2cdf | A | B |
| 1nfd | A | B |
| 2nx5 | N | P |
| 3he6 | C | D |
| 3mff | A | B |
| 3o6f | C | D |
| 1bd2 | D | E |
| 3dx9 | A | B |
| 3mv9 | D | E |
| 3qiw | C | D |
| 3rug | E | F |
| 3c6l | A | B |
| 3pqy | D | E |
| 2cdg | A | B |
| 2esv | D | E |
| 3rgv | A | B |
